# Social Isolation, Related Constructs and Problematic School Attendance Problems in Adolescence: A Systematic Review

**DOI:** 10.3390/children13081020

**Published:** 2026-07-31

**Authors:** Giuseppe Accogli, Giorgia Carlucci, Antonio Trabacca, Maria Gloria Rossetti, Sara Scoditti, Paolo Brambilla, Roberto Sassi, Antonella Delle Fave, Cinzia Bressi, Fabrizia Claudia Guarnieri, Isabella Fanizza, Marcella Bellani

**Affiliations:** 1 Scientific Institute IRCCS “E. Medea”, Scientific Direction, 23842 Bosisio Parini, Italy; giuseppe.accogli@lanostrafamiglia.it; 2 Unit for Severe Disabilities in Developmental Age and Young Adults (Developmental Neurology and Neurorehabilitation), Scientific Hospital for Neurorehabilitation, Associazione “La Nostra Famiglia”, Scientific Institute IRCCS “E. Medea”, 72100 Brindisi, Italy; giorgia.carlucci@lanostrafamiglia.it (G.C.); sara.scoditti@lanostrafamilgia.it (S.S.); isabella.fanizza@lanostrafamiglia.it (I.F.); 3 Section of Psychiatry, Department of Neurosciences, Biomedicine and Movement Sciences, University of Verona, 37134 Verona, Italy; mariagloria.rossetti@univr.it (M.G.R.); marcella.bellani@univr.it (M.B.); 4 Unit of Psychiatry, Azienda Ospedaliera Universitaria Integrata (AOUI) Verona, 37126 Verona, Italy; 5 Department of Pathophysiology and Transplantation, University of Milan, 20122 Milan, Italy; paolo.brambilla1@unimi.it (P.B.); antonella.dellefave@unimi.it (A.D.F.); cinzia.bressi@unimi.it (C.B.); 6 Department of Neurosciences and Mental Health, Fondazione IRCCS Ca’ Granda Ospedale Maggiore Policlinico, 20122 Milan, Italy; 7 Department of Computer Science, University of Milan, 20133 Milan, Italy; roberto.sassi@unimi.it; 8 CNR Institute of Neuroscience, 20854 Vedano al Lambro, Italy; fabrizia.guarnieri@in.cnr.it

**Keywords:** adolescence, social disconnection, social isolation, loneliness, problematic school attendance, school refusal, absenteeism, school dropout, peer rejection, school belonging

## Abstract

**Highlights:**

**What are the main findings?**

• Loneliness, bullying, and school alienation track with school refusal and absenteeism; peer acceptance, reciprocal friendships, and teacher support track with graduation and successful school transition.
• Longitudinally, loneliness and weak teacher support precede intentions to quit school, while supportive relationships buffer against later disengagement.

**What are the implications of the main findings?**

• Attendance problems may benefit from relational, not only symptomatic, assessment; the evidence reviewed suggests that school connectedness could represent a promising, potentially modifiable target for future prevention and intervention efforts.
• The field needs longitudinal, multi-informant studies that separate subjective, interpersonal, and institutional disconnection, and distinguish school refusal, truancy, withdrawal, and dropout.

**Abstract:**

**Background**: Problematic school attendance in adolescence is a heterogeneous phenomenon with academic, emotional, and developmental consequences. Although relational factors are increasingly recognised, the literature remains conceptually fragmented, with overlapping constructs such as social isolation, loneliness, social withdrawal, and peer difficulties examined under partially distinct frameworks. **Objective**: This systematic review aimed to synthesize empirical evidence on the association between social isolation, related constructs, and problematic school attendance during adolescence. **Methods**: Following PRISMA 2020 guidelines, searches were conducted in PubMed, Scopus, and Embase (final search: 20 January 2026). Eligible studies were observational or qualitative empirical investigations involving adolescents aged 11–18 years and examining at least one relational construct (objective/subjective social isolation, loneliness, social withdrawal, peer rejection) together with at least one school attendance outcome (school refusal, withdrawal, truancy, avoidance, dropout). Risk of bias was assessed with design-specific JBI tools and findings were synthesized narratively. **Results**: Eleven studies met the inclusion criteria: six cross-sectional, four longitudinal/cohort, and one qualitative. Loneliness, bullying, school alienation, and social isolation at school were most consistently associated with school refusal and absenteeism, whereas peer acceptance, reciprocal friendships, teacher emotional support, and school belonging were associated with reduced dropout intentions, higher graduation rates, and better school transition. Longitudinally, loneliness and low teacher support preceded increases in intention to quit, while supportive relationships appeared to protect against later disengagement. **Conclusions**: Social isolation and related constructs are meaningfully associated with problematic school attendance in adolescence. Social isolation and related constructs appear to be meaningfully associated with problematic school attendance in adolescence, although the evidence base remains limited and heterogeneous. These findings suggest that a relational perspective may usefully complement existing approaches to attendance difficulties, and point to the need for longitudinal, multi-informant studies disentangling subjective, interpersonal, and institutional disconnection across distinct attendance profiles.

## 1. Introduction

There are several ways to conceptualize the condition in which an individual has limited, unsatisfactory, or perceived-as-absent social relationships, or feels that they do not truly belong to a social network. Social disconnection, therefore, concerns not only the quantity of social contacts, but also the quality of relationships and the subjective sense of belonging, and may consequently take different forms [1].

For example, Social isolation (SI) broadly refers to a condition characterized by limited social contact and reduced community engagement [2,3]. A useful distinction can be drawn between objective and subjective forms of social isolation [4,5,6,7]. Objective social isolation refers to the tangible aspects of isolation, such as physical separation and the absence or infrequency of interactions with others. To assess objective social isolation, individuals are often asked to report the size of their social network, the frequency of interactions with network members, and their level of participation in groups, volunteering, or other social activities and events [8]. By contrast, subjective social isolation is defined by the perceived quality of an individual’s relationships with network members, as well as perceived integration and involvement within social networks [7]. This distinction is especially relevant in adolescence, when the subjective meaning of peer relationships and social belonging may be as important as the objective quantity of social contact. Within this broader framework, loneliness represents a related but distinct construct [9].

Loneliness is generally understood as the subjective experience of dissatisfaction with one’s social relationships, or the perceived discrepancy between desired and actual social connectedness [4,5,10]. In adolescence, loneliness is particularly relevant because social belonging, peer acceptance, and integration into school-based social networks become central developmental tasks [11,12,13]. At this developmental stage, school is not simply an educational setting: it is also a primary relational environment in which adolescents negotiate status, belonging, friendship, exclusion, and recognition. For this reason, loneliness during adolescence may have implications not only for emotional well-being but also for school engagement and persistence. During the school years, the intensity of experienced loneliness is associated with sleep problems [14], poor academic outcomes [15], and school refusal [16]. Sleep disturbances can exacerbate feelings of school refusal both directly and indirectly, with loneliness acting as a mediating factor in this relationship [14]. Several studies have also shown that limited social interaction can adversely affect mental health [6,17]. More specifically, social isolation during adolescence has been associated with psychological health problems, including depression [18,19,20]. Adolescents’ perceived experiences of peer exclusion, such as not belonging to a peer group or “being an outsider,” have been linked to emotional distress, as well as feelings of hopelessness and sadness [21]. These relational experiences may also be relevant to adolescents’ engagement with school, as school attendance is embedded within a broader interpersonal context involving peers, teachers, and feelings of belonging or exclusion. Accordingly, school attendance problems should not be conceptualised solely as behavioural or administrative phenomena, but also as experiences potentially shaped by adolescents’ relational position within the school context.

Also social withdrawal can be considered a phenomenon within the broader domain of social isolation rather than a synonymous construct. Specifically, social withdrawal refers to the deliberate avoidance of, or disinterest in, social activities and interactions [22]. Compared with their peers, socially withdrawn adolescents are more likely to exhibit lower academic achievement, poorer self-efficacy and self-esteem, and higher levels of depression, social anxiety, and suicidal ideation [23,24,25,26]. Socially withdrawn youth are also at increased risk for adjustment problems in adulthood [27]. Although social withdrawal is conceptually distinct from social isolation and loneliness, these constructs may overlap in meaningful ways when adolescents progressively disengage from peers and school life.

A more severe form of prolonged social withdrawal, Hikikomori—voluntary, persistent withdrawal from social relationships, often at home for extended periods [28,29]—typically emerges during adolescence and is associated with elevated psychopathological symptoms [30,31,32,33]. It is conceptually distinct from loneliness or routine absenteeism and is considered here only insofar as it helps frame extreme forms of social withdrawal overlapping with school disengagement.

A growing body of empirical work has examined the association between relational variables and school attendance difficulties during adolescence [34,35,36,37,38,39,40,41,42,43].

Taken together, this literature suggests that problematic school attendance may be associated not only with individual vulnerability, but also with adolescents’ experience of belonging, exclusion, and support within the school environment. In particular, peer acceptance and reciprocal friendships among classmates [44,45,46] are critical factors that may not only predict school dropout, but also help explain why students are absent, achieve low grades, and display academic disengagement. Likewise, objective social isolation or the perception of being excluded from the peer group may mark the beginning of progressive disengagement from the school context. Specifically, when adolescents perceive themselves as marginalised or as not belonging to their peer group, they may experience negative emotions such as anxiety, shame, or sadness [47,48,49]. Over time, these experiences may foster negative expectations toward school, contributing to avoidant behaviours and gradual school withdrawal [43]. This conceptualization suggests that school non-attendance may sometimes represent not only a response to academic difficulty or psychopathology, but also a response to an environment experienced as socially threatening, emotionally unrewarding, or chronically excluding.

School represents a central context for development. A young person’s absence from this context may create or exacerbate deviations from normative developmental trajectories and seriously compromise socio-emotional development [50,51,52], potentially contributing to the onset of mental health disorders [53]. School non-attendance negatively affects learning and academic achievement [54,55], increases the risk of substance use [56], early school dropout [57], and unemployment [58]. Moreover, a significant proportion of youth who do not attend school become involved in juvenile delinquency, thereby disrupting their educational trajectories [59]. For these reasons, understanding the relational correlates of school non-attendance is not only theoretically relevant, but also important for prevention and intervention.

There are numerous ways to define absence from school [60]. School attendance problems are heterogeneous in terms of both etiology and presentation [61]. In the literature, different types of school attendance problems have been identified, including School Refusal (SR), Truancy (TR), and School Withdrawal (SW) [61]. This heterogeneity is important because different attendance profiles may be associated with different relational and emotional mechanisms.

SR involves a reluctance to attend an educational setting, accompanied by emotional distress, as well as the youth’s absence from school with the awareness of parents or caregivers, in the absence of severe antisocial behaviors [61]. It is well documented that school refusal negatively impacts young people. International research has identified immediate effects such as school dropout [62], illicit drug use [63], cigarette smoking [64], and mental health difficulties [65]. Long-term effects include violence, persistent mental health problems, and substance abuse [66].

TR has been defined narrowly as the intentional absence from school without parental consent, often concealed, or more broadly as any unexcused absence. In both conceptualizations, unauthorized absences and limited parental monitoring are common [67]. The literature indicates that truant youth exhibit more behavioral problems, academic difficulties, a higher risk of delinquent behaviors, family-related problems, and socio-economic disadvantage [68,69,70].

SW refers to a form of school absenteeism that is predominantly parent-initiated or parent-supported [71,72]. It occurs when the absence is tolerated, encouraged, or justified by parents, sometimes with weak or untruthful explanations [73]. The motivations can be familial (need for help at home, companionship, economic demands), school-related (protection from perceived threats or conflicts with school, incomplete assignments, malnutrition, or punishment), or instrumental/therapeutic, including sabotage of school reintegration interventions [74].

Accordingly, the aim of this review is to examine how social isolation and related constructs are associated with problematic school attendance behaviours, educational disengagement, and broader school persistence outcomes in adolescence.

**Figure 1:**
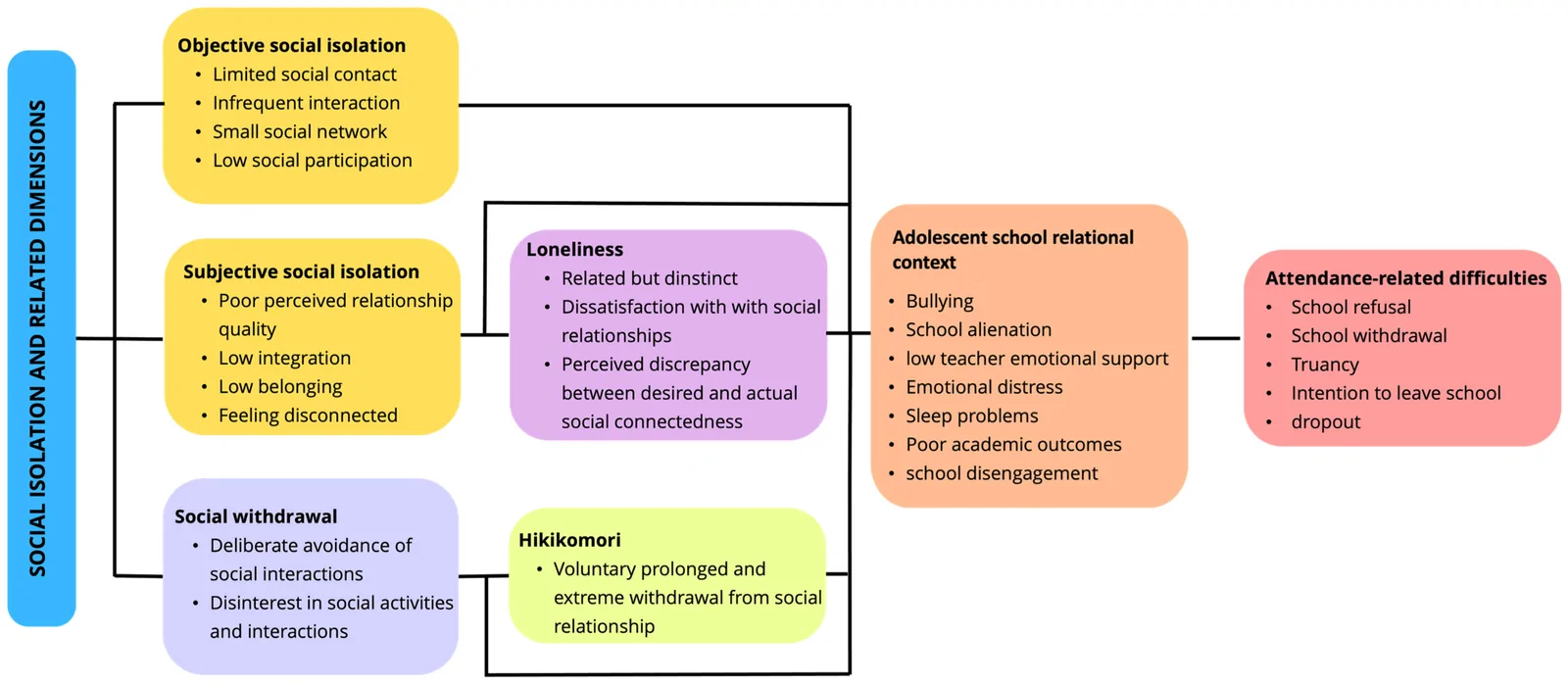
Schematic representation of the main dimensions of social isolation and related constructs, illustrating their connections with the adolescent school relational context and school attendance difficulties.

Figure 1 provides a schematic overview of the key terms used for this purpose.

To improve conceptual clarity, the review focused on social isolation and related dimensions as primary variables of interest rather than on highly specific populations in which isolation is structurally embedded in severe medical, traumatic, or violence-related circumstances. This choice was intended to make the evidence base more comparable, although it necessarily narrows the scope of inference [75]. Overall, the review seeks to provide a more integrated understanding of how different forms of social isolation may contribute to school attendance problems during adolescence, and to clarify whether apparently distinct constructs converge on a broader vulnerability dimension relevant to school disengagement.

## 2. Methods

### 2.1. Design and Scope

This systematic review aimed to synthesize empirical evidence on the association between social isolation, related constructs and school attendance problems during adolescence. Given the conceptual heterogeneity of the literature, the review considered objective and subjective social isolation, loneliness, social withdrawal and peer rejection. A narrative synthesis was conducted to examine how these constructs were associated with school refusal, school withdrawal, truancy-related non-attendance and distal educational outcomes such as dropout. The review was restricted to observational and qualitative empirical studies to focus on non-experimental associations between relational dimensions and school attendance-related outcomes.

### 2.2. Eligibility Criteria

#### 2.2.1. Inclusion Criteria

Studies were included if they met the following criteria:

• Involved adolescents aged 11–18 years, or samples from which adolescent data could be clearly extracted;
• Examined at least one relational construct, including objective and subjective social isolation, loneliness, social withdrawal, peer rejection;
• Examined at least one school attendance-related outcome, including school refusal, school withdrawal, truancy, school avoidance, dropout;
• Were primary empirical studies with an observational or qualitative design;
• Were published in peer-reviewed journals.

#### 2.2.2. Exclusion Criteria

Studies were excluded if they:

• Focused on highly specific subpopulations in which social isolation was primarily linked to severe contextual, traumatic, or medical conditions (e.g., war-related experiences, severe organic illness, hospitalization, or abuse-related circumstances);
• Included exclusively adult samples, exclusively child samples outside the target age range, or mixed-age samples without extractable adolescent data;
• Were intervention studies, including randomised controlled trials;
• Were reviews, meta-analyses, case reports, letters, opinion papers, or book chapters;
• Did not address both a social isolation-related construct and a school attendance-related outcome.

### 2.3. Information Sources and Search Strategy

Searches were conducted in Scopus, Embase, and PubMed. The final search was completed on 20 January 2026. Full search strings and applied filters are reported in Table 1. The search strategy covered two main domains: (1) social isolation and related constructs (e.g., social isolation, social withdrawal, hikikomori, loneliness, peer rejection); and (2) school attendance-related outcomes (e.g., school refusal, school withdrawal, truancy, school phobia, and school dropout). Search syntax was adapted to the requirements of each database. The search was restricted to English-language publications, as English is the predominant language of publication in this research area and this restriction ensured consistent and accurate interpretation of study methods, measures, and findings during screening, data extraction, and quality appraisal, without reliance on translation.

### 2.4. Study Selection

**Figure 2:**
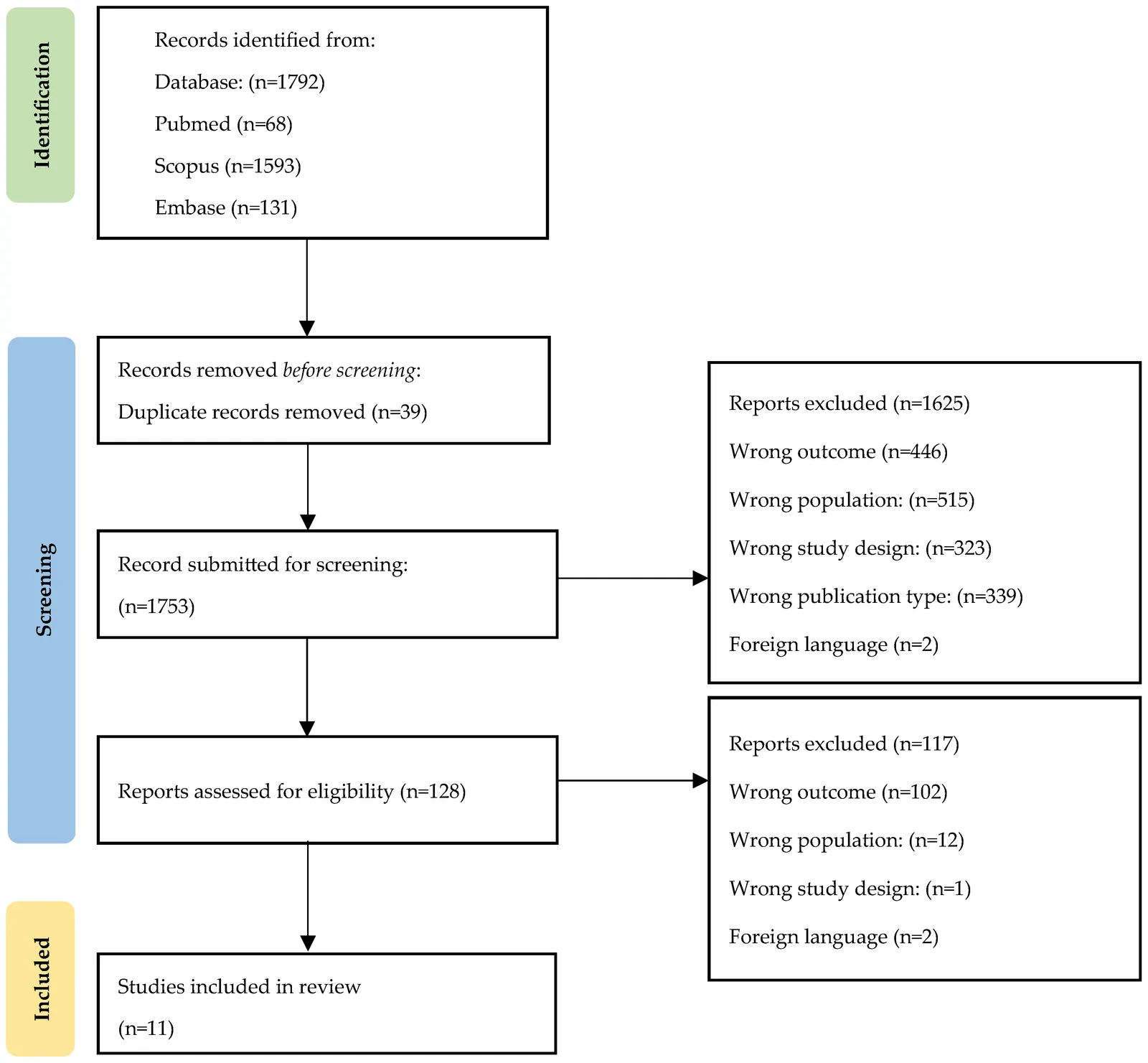
PRISMA Flow diagram.

All retrieved records were imported into a reference management system (Zotero version 9.0.4; https://www.zotero.org/) and duplicates were removed before screening. Titles and abstracts were screened against the predefined eligibility criteria, followed by full-text assessment of potentially relevant articles. Reasons for exclusion were documented, including ineligible population, irrelevant outcome, ineligible study design, ineligible publication type, and non-English language. Two reviewers independently conducted the literature search and screening process, and disagreements were resolved through consultation with a third reviewer. The study selection process was reported using a PRISMA 2020 flow diagram (Figure 2) to enhance transparency and reproducibility [76].

### 2.5. Data Extraction

Data from the included studies were extracted using a structured data extraction form. The following information was collected: authors and year, study aim, study design, sample characteristics, measures and indicators of social isolation-related constructs, school attendance-related outcomes, methods of analysis, and main findings. The extracted data were summarized in Table 2.

### 2.6. Risk of Bias Assessment

**Figure 3:**
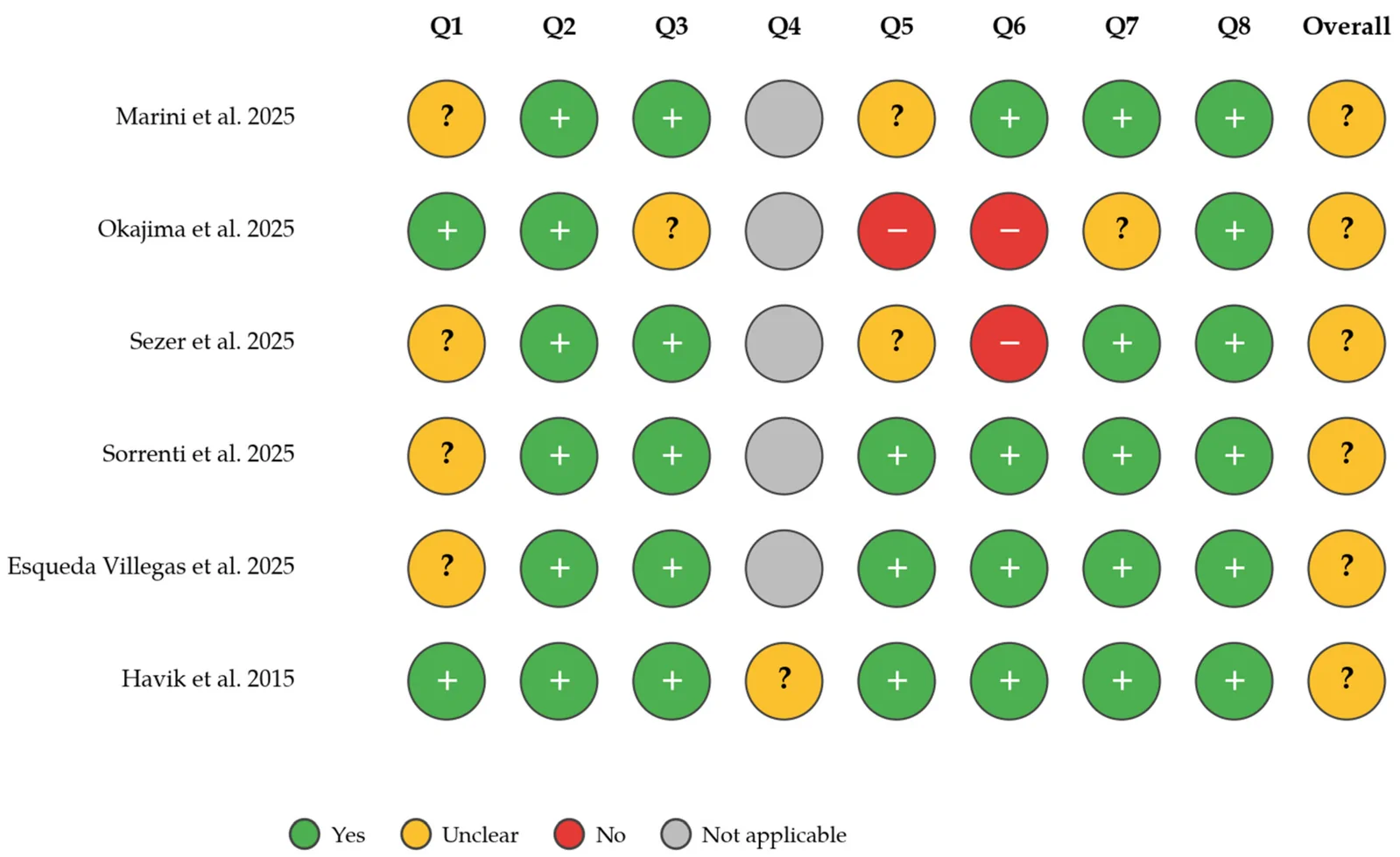
*Analytical Cross-Sectional Studies:* Q1 inclusion criteria defined; Q2 subjects/setting described; Q3 exposure measured validly; Q4 objective/standard criteria for the condition; Q5 confounders identified; Q6 strategies to deal with confounders; Q7 outcomes measured validly; Q8 appropriate statistical analysis. Included studies: Marini et al., 2025 [34]; Okajima et al., 2025 [35]; Sezer et al., 2025 [36]; Sorrenti et al., 2025 [77]; Esqueda Villegas et al., 2025 [37]; Havik et al., 2015 [78].

**Figure 4:**
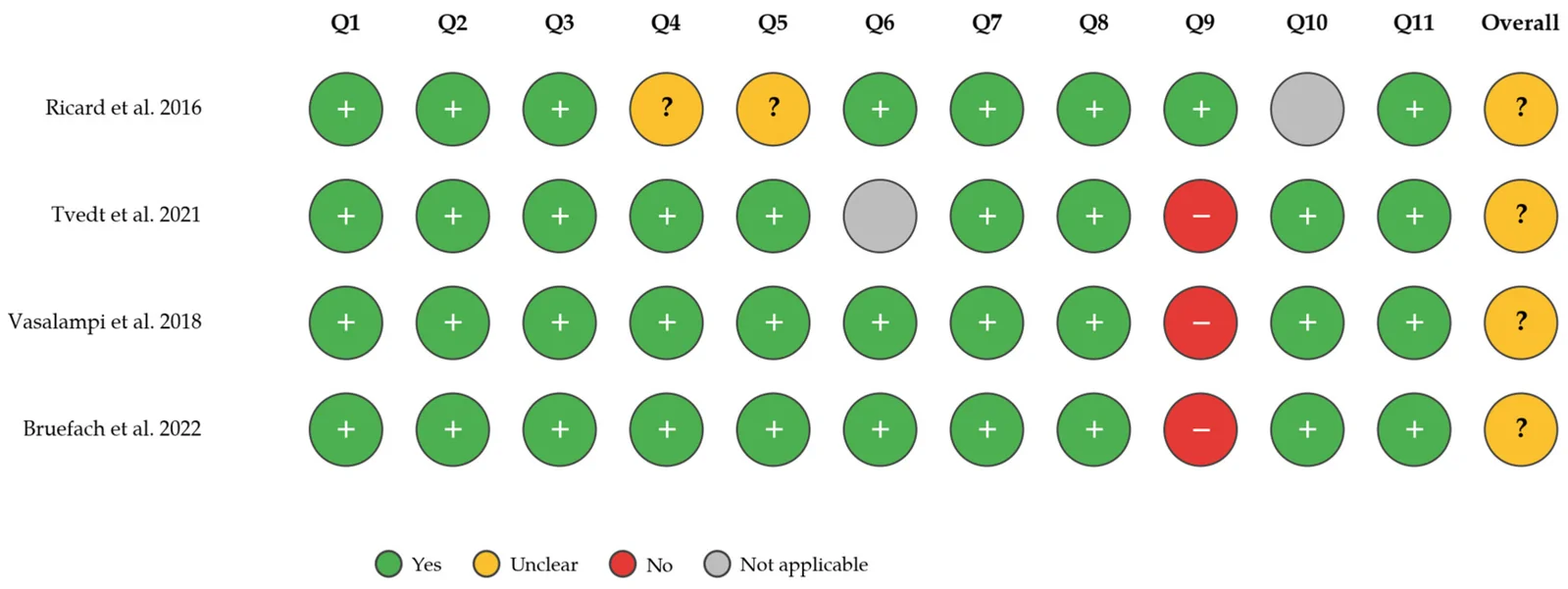
*Cohort Studies*: Q1 groups from same population; Q2 exposures measured similarly across groups; Q3 exposure measured validly; Q4 confounders identified; Q5 strategies to deal with confounders; Q6 free of outcome at baseline; Q7 outcomes measured validly; Q8 follow-up time sufficient; Q9 follow-up complete (or loss explored); Q10 strategies for incomplete follow-up; Q11 appropriate statistical analysis. Included studies: Ricard et al., 2016 [39]; Tvedt et al., 2021 [40]; Vasalampi et al., 2018 [41]; Bruefach et al., 2022 [42].

**Figure 5:**
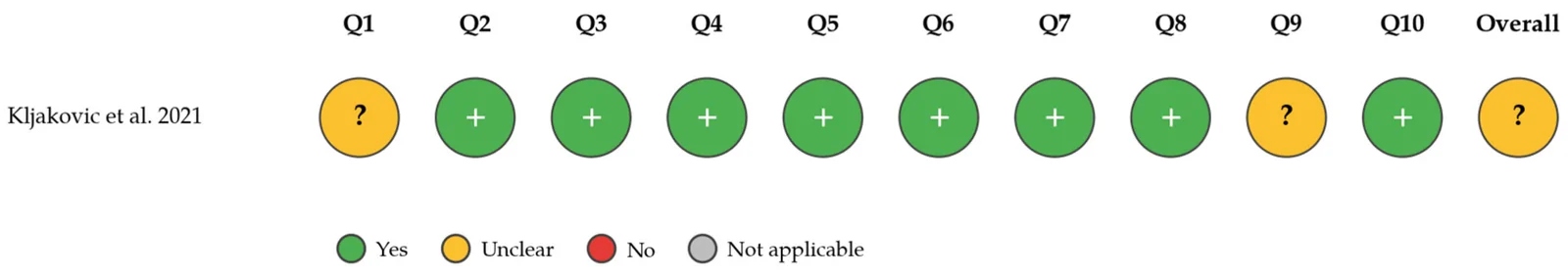
*Qualitative Studies*: Q1 congruity philosophy/methodology; Q2 methodology/research question; Q3 methodology/data collection; Q4 methodology/data analysis; Q5 methodology/interpretation; Q6 researcher located culturally/theoretically; Q7 researcher influence addressed; Q8 participants’ voices represented; Q9 ethical approval; Q10 conclusions flow from analysis. Study included Kljakovic et al., 2021 [43].

Methodological quality was assessed using design-specific Joanna Briggs Institute (JBI) critical appraisal tools. The JBI Analytical Cross-Sectional Studies checklist was used for cross-sectional studies [79], the JBI Cohort Studies checklist for longitudinal/cohort studies [79], and the JBI Checklist for Qualitative Research for the qualitative study [80]. Results are summarised as a traffic-light plot (Figure 3, Figure 4 and Figure 5).

### 2.7. Data Synthesis

The evidence was synthesized by outcome domain, including: (a) intentions to leave school or early school leaving intentions; (b) school refusal and absenteeism, including reasons for non-attendance; and (c) distal educational outcomes, such as dropout, graduation, and school transition. This approach allowed comparison across studies that operationalized social isolation in different but conceptually related constructs.

## 3. Results

The database search identified 1792 records (PubMed n = 68; Scopus n = 1593; Embase n = 131). After removing 39 duplicates, 1753 records were screened by title/abstract and 1625 were excluded. Of 128 full-text reports assessed for eligibility, 117 were excluded. Overall, 11 studies met the inclusion criteria and were included in the review (Figure 2, PRISMA flow diagram).

Eleven studies were included: six cross-sectional surveys, four longitudinal/cohort studies, and one qualitative interview study. Across the quantitative studies, social isolation was operationalised through loneliness/perceived isolation [35,40,42], peer relationship indicators (e.g., bullying, social isolation at school, peer acceptance, reciprocal friendships; [39,41,78]), and school belonging-related constructs (e.g., marginality, alienation, social exclusion, social inclusion; [34,36,37,77]). Outcomes included intentions to leave school, school refusal/absenteeism indicators, and objective/distal educational outcomes (dropout vs. graduation; post-secondary transition). The qualitative study focused on adolescents’ experiences of school refusal and isolation [43].

### 3.1. Intentions to Leave School/Early School Leaving Intentions

Four studies examined intention to leave school or related dropout cognitions. Marini et al. [34] found that higher perceived classroom marginality was associated with stronger dropout intentions, while teacher–student relationship quality was negatively associated with both marginality and dropout intentions. Their moderated mediation model further suggested that teacher–student relationship quality attenuated the indirect association between environmental sensitivity and dropout intentions via perceived marginality.

Esqueda Villegas and Pozas [37] reported that intention to quit school early was associated with several individual and classroom variables. In bivariate analyses, lower emotional well-being, lower academic self-concept, and lower social inclusion were associated with lower intention to quit, whereas SEN status and perceived differentiated instruction were positively associated with intention to quit. In the multilevel model, higher intention to quit remained associated with SEN status, perceived differentiated instruction, and social inclusion, while emotional well-being showed a protective association.

Tvedt et al. [40], using three-wave longitudinal data, showed that increases in loneliness among peers at school were associated with increases in intentions to quit, whereas increases in teacher emotional support were associated with decreases in intentions to quit over time. Similarly, Vasalampi et al. [41] found that intention to drop out was negatively associated with peer acceptance, parental support, autonomous motivation, goal effort, goal progress, and school satisfaction, and positively associated with controlled motivation. Reported indirect effects suggested that both peer acceptance and parental support were linked to dropout intention through motivational processes.

Taken together, these studies suggest that early school-leaving intentions are associated not only with individual adjustment variables, but also with adolescents’ relational position within the school environment.

### 3.2. School Refusal and Absenteeism (Including Reason for Non-Attendance)

Okajima and Hiruma [35] examined feelings of school avoidance, loneliness, sleep-related variables, and self-reported absences in Japanese adolescents. Feelings of school avoidance were positively associated with loneliness intensity, and absences were associated with school avoidance and chronotype. Cluster analyses further suggested that adolescents with higher loneliness and loneliness orientation showed more sleep debt and higher school avoidance.

Sorrenti et al. [77] found that perceived school atmosphere and school alienation were associated with multiple dimensions of school refusal, including anxious anticipation, difficult transition, interpersonal discomfort, and school avoidance. In particular, more negative interpersonal and school climate variables were associated with higher alienation, which in turn was linked to stronger school refusal dimensions.

Havik et al. [78] reported that bullying, social isolation at school, and teachers’ classroom management were associated with both school refusal-related and truancy-related reasons for non-attendance. In general, bullying and social isolation were positively associated with non-attendance reasons, whereas better classroom management showed negative associations with both school refusal and truancy.

Overall, these studies suggest that school refusal and absenteeism are linked to loneliness, alienation, bullying, social isolation, and broader perceptions of the school relational climate.

### 3.3. Objective and Distal Educational Outcomes (Dropout, Graduation, and School Transition)

Ricard and Pelletier [39] followed Canadian Grade 10 students and found that reciprocal friendships and parent needs support were associated with lower likelihood of dropout two years later.

Bruefach and Reynolds [42], using Add Health data, reported that several indicators of social isolation were associated with lower likelihood of high school graduation. In particular, having more friends and higher friends’ educational expectations were associated with higher probability of graduating, whereas higher perceived isolation was associated with lower chance of graduating. Social isolation measures also mediated part of the association between learning disability status and later graduation.

Vasalampi et al. [41] extended their model to successful transition beyond upper secondary education and found that lower intention to drop out predicted better transition outcomes. Indirect effects further suggested that goal progress and paternal support contributed to successful transition through reduced dropout intention.

Together, these studies indicate that social disconnection—a broad umbrella term encompassing distinct, non-interchangeable dimensions such as loneliness, peer rejection, alienation, and reduced belonging—is associated not only with proximal school attendance difficulties, but also with more distal educational outcomes such as dropout, graduation, and post-school transition.

### 3.4. Qualitative Findings

Kljakovic et al. [43] conducted semi-structured interviews with five adolescents enrolled in an Individual Tuition programme after prolonged absence from mainstream education. Thematic analysis identified six themes: isolation, reasons for school refusal, internet use, Individual Tuition, current situation, and values. Participants described extensive time spent isolated at home, variability in social contact, anxiety/social anxiety and sleep difficulties as barriers to school attendance, and frequent reliance on internet and social media as a primary mode of contact.

These qualitative findings complement the quantitative evidence by illustrating how school refusal may be embedded in broader experiences of isolation, disrupted routines, and limited in-person social connections.

### 3.5. Risk of Bias Assessment

Risk of bias was assessed using design-specific Joanna Briggs Institute (JBI) critical appraisal tools: the JBI Analytical Cross-Sectional Studies checklist for cross-sectional surveys [79], the JBI Cohort Studies checklist for longitudinal/cohort studies [79], and the JBI Checklist for Qualitative Research for the qualitative study [80].

Cross-sectional studies (n = 6). Across cross-sectional studies, descriptions of the sample and setting and the appropriateness of statistical analyses were generally adequate. Common concerns related to confounding, with several studies providing limited identification of potential confounders and/or limited strategies to address them, often relying on bivariate analyses or minimal covariate adjustment. Measurement relied predominantly on self-report instruments, which could represent a potential source of information bias. The item concerning objective diagnostic criteria was frequently not applicable, as outcomes were not based on clinical diagnoses.

Longitudinal/cohort studies (n = 4). Cohort studies generally reported adequate follow-up duration and clearly defined outcomes, including objective ascertainment in at least one study. The main concerns related to incomplete follow-up/attrition in multi-wave designs and, in some cases, exposure measurement limitations (e.g., potential misclassification). All longitudinal studies reported explicit strategies to handle missing data (e.g., FIML or multiple imputation/weights).

Qualitative study (n = 1). The qualitative study showed good congruence between the research aims, interview-based data collection, and thematic analysis, and included participant quotations to support themes. Some items were rated unclear due to limited reporting of philosophical positioning and/or ethics approval details.

Overall, the evidence base was informative but methodologically mixed, particularly with regard to confounding, self-report bias, and the limited capacity of most included studies to support causal inference.

## 4. Discussion

The present systematic review suggests that social isolation with related experiences is meaningfully associated with problematic school attendance during adolescence. Across the 11 included studies, problems in relationships—captured through loneliness, marginality, alienation, peer isolation, reduced peer acceptance, weak reciprocal friendships, and lower school belonging—were linked to school refusal, absenteeism, intention to leave school, lower likelihood of graduation, and poorer transition outcomes. Although the literature used heterogeneous labels and measures, the overall pattern was convergent, supporting the view that different indicators of social disconnection may reflect a broader vulnerability dimension relevant to school disengagement [70].

One plausible interpretation is that social isolation does not merely co-occur with attendance problems, but may contribute to transforming school from a developmental context of participation and recognition into a socially threatening or emotionally unrewarding environment. This interpretation is especially plausible in adolescence, a developmental stage in which peer evaluation, status, and belonging are highly salient, and in which social connectedness has important implications for mental health and adjustment. In this framework, loneliness, exclusion, and alienation may increase anticipatory distress, shame, sadness, and negative expectations about interpersonal encounters at school, thereby fostering avoidance and, over time, disengagement [81,82].

Importantly, the reviewed studies suggest that different experiences of social isolation may not affect all forms of school attendance problems equally. In studies focusing on school refusal and absence-related distress, loneliness, bullying, school alienation, and other indicators of social difficulties appeared especially tied to emotionally driven non-attendance. Although not tested directly within the included studies, this pattern is consistent with broader external evidence showing that anxiety is particularly associated with school refusal, whereas depression appears more clearly linked to absenteeism and truancy. By contrast, in studies examining dropout intentions and distal educational outcomes, peer acceptance, reciprocal friendships, teacher emotional support, and school belonging appeared to serve more as protective resources for persistence, engagement, and successful transition. Thus, social disconnection may be conceptualised both as an affective risk factor for avoidance and as a relational marker of weakened school attachment [83,84].

The longitudinal findings included in this review are particularly informative because they point beyond a purely concurrent association. Although longitudinal evidence was limited, studies following adolescents over time showed that higher loneliness and weaker teacher emotional support were associated with worsening intentions to quit school, while peer acceptance, reciprocal friendships, and supportive relationships were linked to lower dropout risk and better transition outcomes. These findings are consistent with broader longitudinal work indicating that both school and social connectedness in early adolescence are associated with later mental health and school completion. At the same time, the available evidence does not yet allow strong causal conclusions, and bidirectional processes remain likely [84].

A key conceptual implication of this review is that the field may benefit from moving away from the search for a single “best” indicator of social isolation. The included studies assessed loneliness, perceived isolation, school alienation, marginality, social inclusion, peer relationships, and school belonging, often with limited harmonisation across measures. Rather than treating these as unrelated constructs, future work may benefit from a multilevel framework distinguishing between subjective disconnection (e.g., loneliness), interpersonal disconnection (e.g., peer rejection or lack of reciprocal friendships), and institutional disconnection (e.g., alienation from teachers or school). Such an approach may help clarify why some adolescents present primarily with school refusal, whereas others move toward chronic absenteeism or early school leaving. The unexpected positive association between social inclusion and intention to quit observed in one study also underscores the need for more precise measurement and for attention to possible suppression effects or context-specific meanings of inclusion [37]. Beyond the direct evidence synthesised here, these findings also point to broader implications for clinical and school practice, which future studies should test directly. Adolescents with attendance problems might benefit from being assessed not only in terms of individual symptoms or motivation, but also in terms of loneliness, peer exclusion, bullying, weak teacher relationships, and low school belonging. This broader relational assessment may be particularly relevant given prior evidence that school connectedness can act as a protective factor for depression and anxiety [81,82], suggesting—though not directly tested by the included studies—that interventions strengthening school connection could have downstream benefits for emotional wellbeing and school retention. More generally, recent reviews portray school refusal as a multidetermined condition in which relational, emotional, and psychopathological factors intersect, rather than as a unitary attendance problem [66,82,85].

Several limitations should temper interpretation. First, although the review adopted a systematic and transparent approach, its scope was restricted to three databases (PubMed, Scopus, and Embase), to English-language studies, and to publications from 2015 onward; in addition, grey literature and non-empirical publications were excluded by design. While these choices improved methodological consistency, they may also have reduced the coverage of potentially relevant educational or school-psychology literature and may have contributed to the relatively small final evidence base (11 studies). With respect to language, the search was restricted to English-language publications. Overall, only four records were excluded because of this restriction across the whole screening process (two at the title/abstract stage and two at the full-text stage), out of 1742 records excluded in total, indicating that this restriction is unlikely to have materially affected the composition of the evidence base. Future reviews on this topic may nonetheless benefit from including non-English publications to further broaden coverage.

Second, the review necessarily synthesised a conceptually heterogeneous literature. Across included studies, “social isolation” was operationalised through loneliness, perceived isolation, peer difficulties, bullying-related isolation, marginality, alienation, social inclusion, and school belonging, whereas school attendance outcomes ranged from school refusal symptoms and absenteeism to dropout intentions, graduation, and transition outcomes. This heterogeneity prevented quantitative pooling and complicated direct comparison across studies. It is also particularly relevant because school attendance problems are increasingly conceptualised as heterogeneous phenomena that require clearer differentiation across school refusal, truancy, school withdrawal, and related attendance profiles. A related issue concerns measurement: loneliness and related constructs in children and adolescents are themselves assessed with instruments that differ in conceptual focus and psychometric support, further limiting comparability across studies [61,86].

Third, the conclusions of the review are constrained by the quality of the primary studies. Most included studies were cross-sectional, relied predominantly on self-report measures, and often showed limited control for confounding; the longitudinal studies, while more informative, were affected by attrition or possible exposure misclassification. As a result, causal inference remains limited, and reverse or bidirectional associations cannot be ruled out: social problems may contribute to school non-attendance, but non-attendance may also intensify loneliness, peer estrangement, and alienation from school. In addition, the review included only one qualitative study; therefore the subjective experience of adolescents with attendance difficulties remains underrepresented. Finally, the decision to exclude highly specific subpopulations and context-bound forms of isolation improved comparability, but it also limits the generalisability of the findings to adolescents for whom social disconnection may be especially shaped by trauma, medical conditions, discrimination, or severe psychosocial adversity [87].

Future studies should move beyond broad correlational approaches and adopt designs capable of clarifying temporal ordering and mechanisms. Longitudinal, multi-informant studies are especially needed, combining adolescent self-report with teacher ratings, peer-based indicators, and objective school records of attendance, absenteeism, and dropout. This would help disentangle whether loneliness and peer estrangement precede school attendance problems, emerge as their consequence, or become embedded in reciprocal feedback loops over time. At the conceptual level, future research should more clearly distinguish between subjective loneliness, objective social isolation, peer rejection, social withdrawal, and school belonging, rather than treating them as interchangeable indicators of the same construct. Likewise, school attendance outcomes should be differentiated more consistently across school refusal, truancy, school withdrawal, and dropout, in line with contemporary frameworks on school attendance problems. Greater harmonisation of constructs and measures would substantially improve comparability across studies and may eventually make meta-analytic synthesis feasible [61,86].

Future work should also place greater emphasis on underlying mechanisms. In particular, studies should test which factors mediate or moderate the association between social isolation and related experiences and school non-attendance problems.

These pathways are plausible in light of both the studies included in the present review and the broader literature linking anxiety and school attendance problems, as well as school connectedness with later mental health and educational outcomes. Finally, the field would benefit from more intervention-oriented and mixed-method research. Recent reviews suggest that school-connectedness interventions are promising, but also note ongoing inconsistency in definitions, measures, and intervention targets, with organizational-level approaches still less developed than individual- and interpersonal-level strategies. More qualitative studies are likewise needed to capture adolescents’ own accounts of social disconnection, avoidance, and reintegration into school, since these perspectives may clarify processes that are difficult to capture through questionnaires alone [83,84,87,88,89]. Accordingly, future research should also aim to adopt standardized, consensus-based definitions and operationalizations of the key constructs (e.g., social isolation, school connectedness, belonging), since terminological and measurement heterogeneity currently limits the comparability of findings across studies.

### Clinical and Educational Implications

Building on the findings of this review, several concrete implications can be drawn for clinical and educational practice. In terms of variables to measure, professionals working with adolescents at risk of attendance problems should routinely assess: (a) subjective loneliness and perceived social isolation, using validated adolescent self-report scales [86]; (b) peer relationship quality, including experiences of peer rejection, exclusion, or bullying victimization; (c) school belonging and connectedness, via established school-connectedness measures [82,84]; and (d) the quality of student–teacher relationships, as a further dimension of institutional disconnection [78].

These assessments are particularly relevant within specific educational contexts: during key school transitions (e.g., primary-to-secondary) [84], at the emergence of early, non-chronic absenteeism, and in classrooms where bullying or peer marginalisation has already been identified—moments when relational risk factors are most likely to translate into attendance difficulties.

The preventive rationale is that loneliness, peer rejection, and low school belonging are potentially modifiable, unlike many individual- or family-level risk factors [82]. Early identification would allow schools to intervene through targeted strategies such as peer-support programmes and structured social-skills groups [88,89], before social disconnection consolidates into chronic non-attendance, emotional distress, or dropout.

In conclusion, the available evidence suggests that social isolation and related dimensions are not peripheral to school attendance problems in adolescence, but are instead closely interwoven with emotional distress, school disengagement, and educational persistence. A more relationally informed understanding of attendance problems may therefore improve both conceptual models and early preventive responses [66,84]. These conclusions should nonetheless be interpreted cautiously in light of the limited number and heterogeneity of the included studies.

## Figures and Tables

**Table 1:** Search queries used for each database with the corresponding filters applied. The asterisk (*) indicates truncation, used to retrieve all possible word endings (e.g., teen retrieves teen, teens, teenager, teenagers).

Database	Search Query	Filters
**Scopus**	(‘social isolation’ OR ‘social withdrawal’ OR hikikomori OR loneliness OR ‘peer rejection’ OR isolation AND ‘school refusal’ OR ‘school withdrawal’ OR ‘school absenteism’ OR ‘truancy’ OR ‘school phobia’ OR ‘school dropout’ AND adolescent OR adolescence OR teen OR teenager) AND PUBYEAR > 2014 AND PUBYEAR < 2027 AND (LIMIT-TO (LANGUAGE, “English”))	Language: English All Fields Last 10 years
**PubMed**	((“social isolation” OR “social withdrawal” OR hikikomori OR loneliness OR “peer rejection” OR isolation) AND (“school refusal” OR “school withdrawal” OR “school absenteism” OR truancy OR “school phobia” OR “school dropout”) AND (adolescent OR adolescence OR teen OR teenager))	Language: English All Fields Last 10 years
**Embase**	(‘social isolation’/exp OR ‘social withdrawal’/exp OR ‘hikikomori’/exp OR ‘loneliness’/exp OR ‘peer rejection’/exp OR ‘isolation’/exp) AND (‘school refusal’/exp OR ‘school withdrawal’ OR ‘school absenteeism’/exp OR ‘truancy’/exp OR ‘school phobia’/exp OR ‘school dropout’/exp) AND (‘adolescent’/exp OR ‘adolescence’/exp OR teen* OR teenager*) AND [english]/lim AND [2015–2026]/py	Language: English All Fields Last 10 years

**Table 2:** Characteristics and main findings of the included studies.

Authors	Aim	Experimental Group(s)	Control Group(s)	Measures/Instruments	Outcome Measures	Methods	Main Results
**Marini et al. (2025)** [34]	To test whether environmental sensitivity (ES) is indirectly associated with dropout intentions through perceived marginality, and whether teacher–student relationship quality (TSR) moderates these associations.	Secondary school students in Italy (overall sample n = 744; complete cases n = 736; mean age 16.72 years, range 14–22).	Not applicable (observational study with no intervention/comparator arm).	Highly Sensitive Child Scale (HSC, 12 items); marginality scale (5 items, adapted); dropout intentions scale (3 items, adapted); Teacher–Student Relationship scale (9 items).	Dropout intentions (primary outcome) and classroom marginality (mediator), with ES as predictor and TSR as moderator.	Cross-sectional school survey; correlational analyses; moderation; moderated mediation.	ES predicted higher marginality only at low TSR; marginality predicted higher dropout intentions; the indirect effect ES → marginality → dropout intentions were significant only at low TSR; no significant direct ES → dropout intentions effect.
**Okajima & Hiruma (2025)** [35]	To (1) develop a Loneliness Orientation Test (LOT), (2) examine associations among loneliness intensity/orientation, sleep problems, school refusal feelings, and absenteeism, and (3) test combined loneliness profiles (intensity × orientation) in relation to sleep and school-refusal indicators.	Senior high school students in Tokyo; 168 recruited, 128 analyzed (mean age 16.55 ± 0.64 years; students with complete data and SDI ≥ 0).	Not applicable (observational study; no intervention/comparator arm).	LOT (developed in this study), Three-Item Loneliness Scale (TILS), Athens Insomnia Scale (AIS), Sleep Debt Index (SDI), Circadian Energy Scale (CIRENS), Feeling of School-Avoidance Scale (FSAS), and number of absences.	Loneliness intensity, loneliness orientation, school-avoidance feelings (school refusal-related), and absenteeism, with sleep indicators (insomnia, sleep debt, chronotype) as key correlates/predictors.	Cross-sectional in-person survey; EFA/CFA for LOT structure, correlation analyses, GLMs for associations between sleep/loneliness/school-refusal outcomes, and hierarchical cluster analysis for loneliness profiles.	AIS was associated with loneliness intensity (TILS); TILS and AIS were associated with school-avoidance feelings (FSAS); FSAS, SDI, and CIRENS were associated with absences. Cluster analyses suggested different loneliness-orientation profiles, and lonely adolescents with positive loneliness orientation showed less unfavorable sleep patterns than those with negative orientation.
**Ricard & Pelletier (2016)** [39]	To examine whether parent/teacher support for basic psychological needs, reciprocal friendships, and academic motivation in Grade 10 predict high-school dropout two years later (Grade 12).	Grade-10 students from nine French-speaking schools in one Canadian school district (N = 624; mean age = 15; followed to Grade 12).	Not applicable (observational longitudinal study; no intervention/comparator arm).	Interpersonal Behavior Questionnaire (short version) for parent/teacher support of autonomy, competence, relatedness; sociometric nominations for reciprocal friendships (best-friend nominations); academic Motivation Scale for High School + Self-Determination Index (SDI); school-board academic status at 2-year follow-up (graduated vs. dropped out).	Academic motivation (SDI) and high-school dropout (yes/no); key predictors include reciprocal friendships and parent/teacher need-support.	Prospective longitudinal design (Grade 10 baseline, Grade 12 outcome); hierarchical linear regression for academic motivation and hierarchical logistic regression for dropout.	Reciprocal friendships significantly predicted academic motivation (above parent/teacher support) and significantly predicted lower odds of dropout. Parent support was also a strong negative predictor of dropout; teacher support and academic motivation were not significant dropout predictors in the final model.
**Sezer & Gürtepe (2025)** [36]	To investigate relationships between attachment (mother/father/peer), social exclusion, and risky behaviors in adolescents, and to test whether these variables differ by gender.	High-school adolescents in Turkey (N = 463; age 13–18; grades 9–12).	Not applicable (observational cross-sectional study; no intervention/comparator arm).	Parent and Peer Attachment Inventory; Ostracism (Social Exclusion) Scale for Adolescents; Risky Behaviors Scale (RSBS; includes school dropout subscale).	Risky behaviors (including school dropout), and their associations with attachment and social exclusion.	Cross-sectional relational survey; Pearson/Spearman correlations; group comparisons via t-tests, Mann–Whitney U, ANOVA/Kruskal–Wallis (as appropriate).	Social exclusion showed weak but significant positive associations with school dropout (and also with antisocial behavior/suicidal tendency), while attachment dimensions were negatively associated with several risky behaviors, including school dropout.
**Sorrenti et al. (2025)** [77]	To test (via SEM) whether school alienation (learning, teacher, classmate alienation) mediates associations between perceived school atmosphere dimensions and school refusal dimensions.	528 Italian high-school students, age 14–20 (mean ≈16; mixed school tracks).	Not applicable (observational, cross-sectional design; no intervention/comparator arm).	Multidimensional School Climate Questionnaire—School Atmosphere subscale (MSCQ-SAt); School Alienation Scale (SALS); SChool REfusal EvaluatioN (SCREEN); demographic questionnaire.	School refusal dimensions: anxious anticipation, difficult transition, interpersonal discomfort, school avoidance. Mediator: school alienation dimensions. Predictors: school atmosphere dimensions (student relations, student–teacher relations, educational climate, belonging, interpersonal justice).	Cross-sectional school survey; latent-variable SEM with parceling; bootstrap (5000 samples) for indirect effects; model controlled for age and gender.	Negative perceptions of interpersonal justice and sense of belonging were linked to higher alienation (especially learning/teacher alienation). Alienation dimensions were associated with higher school-refusal components (notably anxious anticipation and difficult transition). Significant indirect (mediated) paths supported a SAt → SAl → SR mechanism.
**Tvedt et al. (2021)** [40]	To examine (1) developmental trajectories of intentions to quit school, perceived emotional support from teachers, and loneliness among peers, and (2) whether initial levels and changes in emotional support/loneliness are associated with change in intentions to quit school.	Upper secondary students in Norway (N = 1379 at baseline; mean age 16.5; 54% vocational track; 17% immigrant background), followed across 13 months (3 waves).	Not applicable (observational longitudinal study; no intervention/comparator arm).	Intentions to quit school scale (5 items); Perceived emotional support from teachers scale (5 items); Loneliness among peers at school (Norwegian version of Loneliness and Social Dissatisfaction Questionnaire; 6 items); Registry covariates: gender, study track, previous academic achievement.	Primary outcome was change in intentions to quit school; key psychosocial correlates were trajectories of teacher emotional support and peer loneliness.	3-wave longitudinal design (T1, T2, T3); latent growth curve modeling (unconditional and multivariate models), FIML for missing data, controls for gender/study track/previous achievement, robust estimation.	Average increase over time in intentions to quit school. Average increase in loneliness among peers. No average change in perceived teacher emotional support. Change in teacher support was negatively associated with change in intentions to quit. Change in loneliness was positively associated with change in intentions to quit. Initial levels of support/loneliness did not significantly predict change in intentions in the multivariate model.
**Vasalampi et al. (2018)** [41]	To examine how supportive interpersonal factors (mother/father affective support, peer acceptance) and education-related goal motivation predict school satisfaction, intentions to drop out, and later success in transition beyond upper secondary education.	Finnish upper-secondary students; N = 1520 at baseline (mean age ~16), followed at three waves (T2: n = 1017; T3: n = 494).	Not applicable (observational longitudinal design; no intervention/comparator arm).	Peer acceptance via sociometric nominations; maternal/paternal affective support (Child Rearing Practices Report, Finnish version); autonomous/controlled goal motivation, goal effort, goal progress (education-related goal appraisal items); school satisfaction scale; intention to drop out scale (2 items); transition success indicator at follow-up.	School satisfaction, intention to drop out, and successful transition beyond upper secondary school; motivational mediation pathways.	3-wave longitudinal survey; path modeling/SEM in Mplus with FIML and robust SE; indirect effects tested (model indirect).	Autonomous motivation → greater goal effort → greater goal progress. Mother support and peer acceptance predicted higher autonomous motivation. Father support and peer acceptance were linked to higher school satisfaction and lower intentions to drop out. Lower intention to drop out predicted more successful transition beyond upper secondary education. Low peer acceptance (social isolation) predicts a higher intention to drop out. Peer acceptance reduces dropout intention by supporting autonomous motivation.
**Bruefach, T.; Reynolds, J. R. (2022)** [42]	To examine multiple dimensions of school-based social isolation among students with learning disabilities (LDs) and test how much these dimensions mediate the LD gap in high school graduation.	Students with LDs (parent-reported) in grades 7–12 from Add Health; analytic sample N = 7907; LD group n = 842.	Students without LDs (same dataset); n = 7065.	Social capital: number of school friends; friends’ educational expectations. Social disconnectedness: avoided/rejected/disinterested categories based on friendship nominations. Perceived isolation scale (includes loneliness and school detachment items). Outcome: high school diploma status by follow-up.	High school graduation (diploma vs. no diploma) and multidimensional social isolation indicators (network size, friends’ expectations, disconnectedness types, perceived isolation/loneliness).	Add Health Waves 1–2–4; regression models + KHB mediation to estimate indirect effects of isolation dimensions on LD–graduation gap; controls include GPA and sociodemographics; missing handled via multiple imputation/FIML (as reported).	LD students showed fewer friends, lower friends’ expectations, higher likelihood of avoidant/disinterested disconnectedness, and higher perceived isolation. Social isolation dimensions jointly explained ~23.5% of the LD gap in high school graduation; the largest contribution came from number of friends and friends’ expectations, with perceived isolation also contributing.
**Esqueda Villegas, F.; Pozas, M. (2025)** [37]	To test whether sociodemographic factors, perception of inclusion (incl. social inclusion), differentiated instruction practices, and general self-efficacy predict students’ intentions to leave school early (ESL intention).	Mexican middle-school students (N = 602; mean age 13.20; grades 6–9; private schools; 34% in inclusive classes; 15% self-reported SEN).	Not applicable (observational cross-sectional study; no intervention/comparator arm).	Intention to Quit Scale (3 items); Perceptions of Inclusion Questionnaire—student version (social inclusion, emotional well-being, academic self-concept); General Self-Efficacy Scale; students’ rating of teachers’ Differentiated Instruction (adapted Teacher Differentiated Teaching Scale).	Primary outcome: intention to quit school early. Key “isolation-related” aspect: social inclusion (inverse of social isolation).	Quantitative cross-sectional survey administered at school; descriptive + correlations; multilevel regression (students nested in classrooms) with predictors at student level and classroom setting (inclusive vs. regular).	Overall intention to quit was low. Higher intention to quit was associated with SEN status; higher emotional well-being and higher academic self-concept correlated with lower intention; (in their models) social inclusion and perceived DI showed significant associations with intention to quit; classroom setting not associated.
**Havik, T.; Bru, E.; Ertesvåg, S. K. (2015)** [78]	To examine how students’ perceptions of peer relationships (bullying, social isolation) and teachers’ classroom management are associated with school refusal-related reasons and truancy-related reasons for non-attendance, controlling for emotional stability and parental variables; and test differences between primary vs. secondary school.	Students (Norway) grades 6–10 (ages 11–15). Total sample N = 5465; analytic subsample = students absent at least once in past 3 months N = 3629.	Not applicable (observational SEM; no intervention/comparator arm). (They do compare primary vs. secondary school levels within the model).	SR-related reasons (4 items); Truancy-related reasons (4 items); Socially isolated at school (5 items incl. “I sometimes feel lonely at school”); Being bullied (4 items); Teachers’ classroom management (2nd-order: emotional/academic support, monitoring, follow-up of non-attendance, predictability); Controls: Emotional stability (JEPQR-S neuroticism items), parental interest in schoolwork, parental monitoring of reasons for absence.	Outcomes: SR-related reasons and truancy-related reasons for school non-attendance; focal predictor here: social isolation/loneliness at school (and bullying), plus teacher classroom management.	Cross-sectional web survey (2012) in 45 schools; SEM with latent variables; multi-group (primary vs. secondary), MLR robust estimation; students with no absence in last 3 months excluded; model estimates total/indirect effects (e.g., teacher management → bullying/isolation → SR reasons).	Bullying showed a strong association with SR reasons (stronger in primary) and a moderate association with truancy reasons. Social isolation showed a moderate association with SR reasons among secondary students and mainly indirect links via bullying. Teachers’ classroom management related indirectly to SR/truancy through reduced bullying/social isolation; in secondary school it also showed direct negative links with SR/truancy reasons.
**Kljakovic, M.; Kelly, A.; Richardson, A. (2021)** [43]	To explore the experiences and views of adolescents with school refusal who were extremely socially withdrawn, focusing on perceived causes, impact of isolation, current life evaluation, and personal values.	Adolescents with school refusal enrolled in an Individual Tuition (IT) programme within a London Pupil Referral Unit (PRU); n = 5, age 13–16, 1 female/4 male; out of mainstream education for ≥6 months.	None (qualitative study; no comparator arm).	Semi-structured interview schedule; audio-recorded interviews; verbatim transcripts.	Perceived pathways to school refusal/withdrawal; isolation/social withdrawal (time alone, social contact); impact of isolation; role of anxiety and sleep; internet/social media as protective/risk factor; values and perceived supports (e.g., IT).	Semi-structured qualitative interviews (18–32 min) conducted mainly at participants’ homes; thematic analysis (Braun & Clarke approach).	Six core themes identified (incl. isolation, reasons for school refusal, internet use, individual tuition, current situation, values). Anxiety and sleep difficulties emerged as key factors; some social contact (including via social media) appeared potentially protective against negative impacts of withdrawal for several participants.

## Data Availability

Data will be available on request to the corresponding author.
